# The genome sequence of the Brown Bagworm, *Taleporia tubulosa* (Retzius, 1783) (Lepidoptera: Psychidae)

**DOI:** 10.12688/wellcomeopenres.25052.1

**Published:** 2025-10-21

**Authors:** David C. Lees, Finlay McIntosh, Danaë Vassiliades, Maxwell V. L. Barclay, Natural History Museum Genome Acquisition Lab, Darwin Tree of Life Barcoding Collective, Wellcome Sanger Institute Tree of Life Management, Samples and Laboratory team, Wellcome Sanger Institute Scientific Operations: Sequencing Operations, Wellcome Sanger Institute Tree of Life Core Informatics team, Tree of Life Core Informatics collective, Darwin Tree of Life Consortium

**Affiliations:** 1 Natural History Museum, London, England, UK

**Keywords:** Taleporia tubulosa; Brown Bagworm; genome sequence; chromosomal; Lepidoptera

## Abstract

We present a genome assembly from an individual male *Taleporia tubulosa* (Brown Bagworm; Arthropoda; Insecta; Lepidoptera; Psychidae). The assembly contains two haplotypes with total lengths of 394.58 megabases and 397.58 megabases. Most of haplotype 1 (99.89%) is scaffolded into 30 chromosomal pseudomolecules, including the Z sex chromosome. Haplotype 2 was assembled to scaffold level. The mitochondrial genome has also been assembled, with a length of 17.11 kilobases. This assembly was generated as part of the Darwin Tree of Life project, which produces reference genomes for eukaryotic species found in Britain and Ireland.

## Species taxonomy

Eukaryota; Opisthokonta; Metazoa; Eumetazoa; Bilateria; Protostomia; Ecdysozoa; Panarthropoda; Arthropoda; Mandibulata; Pancrustacea; Hexapoda; Insecta; Dicondylia; Pterygota; Neoptera; Endopterygota; Amphiesmenoptera; Lepidoptera; Glossata; Neolepidoptera; Heteroneura; Ditrysia; Tineoidea; Psychidae; Taleporiinae; *Taleporia*; *Taleporia tubulosa* (Retzius, 1783) (NCBI:txid753433)

## Background

*Taleporia tubulosa* (Retzius, 1783), also known as the Brown Bagworm, is a moth in the family Psychidae with a forewing length of 7–10 mm ( Sterling *et al*., 2023) or about 15–19 mm wingspan. The wings of the male are a brownish colour with a golden lustre and with darker brown reticulation and the head is covered with a tuft of yellow scales, with pectinate antennae less than half the forewing length; the males fly in May and particularly in June ( Norfolk Moths, 2025), without feeding. The females are yellowish with black head and pronotum and are wingless, larviform, and sit on the larval case for mating.

The yellowish-white ellipsoid eggs are laid among case amongst an aggregation of ochreous scales ( Lepiforum, 2025). The larva feeds on lichens and incorporates fragments of these with other particles such as sand and bark within this silken sac. The larval case is long and tubular, as the species name suggests (triangular in cross-section), about 14–20 mm long ( Sterling *et al*., 2023); the pupal case is extruded on emergence. The cases, easily distinguished by their shape from other Psychidae occurring in Britain, can be found attached to trunks, fences and posts. They overwinter in place.

The moth is widespread in England and Wales occurring south of Manchester and Leeds (384 records on NBN Atlas Partnership, 2025), but is very localised, with a concentration of records around Nottinghamshire; it is apparently absent in Scotland and Ireland. The species is widespread and prevalent across Western Europe, currently with 9 425 records on GBIF, where it occurs from the southernmost Mediterranean coastline to northernmost Scandinavia (note that *Taleporia borealis* (Wocke, 1862) is regarded as a synonym: Arnscheid & Weidlich, 2017) and it ranges east to Central Asia ( GBIF Secretariat, 2025).

The genus *Taleporia* Hübner, 1825 contains some 23 species ( Funet, 2025; Sobczyk, 2011), assuming that *T. politella*, as for *T. borealis*, are valid synonyms of *T. tubulosa*.

On BOLD (22/09/2025) the DNA barcode from the mitochondrion assembly (OZ200956.1) represents the Barcode Index Number (BIN) BOLD:AAC2838, in which the majority of ~47 current exemplars constitute different haplotypes (five in the UK alone). Two other BINs mostly identified as *T. tubulosa*, both widespread in Europe, are BOLD:ADJ5822 (few exemplars), 2.1–2.3% *p*-distant from it, and BOLD:ADK7926 (sometimes identified as *T. politella* (Ochsenheimer, 1816)), which is 1.7–2.9% *p*-distant. In the neighbour-joining tree resulting from a search on BOLD, the sister of these BINs is BOLD:ADK3961 (from Japan, identified as *T. shosenkyoensis* or *T. amariensis*, both Saigusa, 1961), which is about 2.4% *p*-distant.

In Psychidae such as *Taleporia*, the W chromosome is reported to be absent, suggesting two independent origins of this chromosome in Tischeriidae and in advanced Ditrysia to which Psychidae belongs ( Hejničková *et al*., 2019) (see Regier *et al*., 2013 for the position of Psychidae in a Lepidoptera phylogeny). Females lack sex chromatin, have a lower chromosome number and smaller genome size than males. Instead, Psychidae show a Z0/ZZ sex chromosome system. The genome will be useful for further studies in lepidopteran chromosome evolution, as well as studies of winglessness (e.g., Niitsu *et al*., 2017). This detailed phylogenetic study based on 28S rRNA and reconstructing patterns of wing degeneration in the family ( Niitsu *et al*., 2017) recovered *Taleporia* as sister to the genus *Kozhantshikovia* Saigusa, 1961 and the subfamily Taleporiinae as sister to Narycinae.

The specimen was collected during a Natural History Museum entomological survey of Wetherby Gardens, Kensington, organised by Holly Smith and Neil Osborn, NHM patrons and editors of ‘Garden Square News’.

## Methods

### Sample acquisition and DNA barcoding

**Figure 1.**
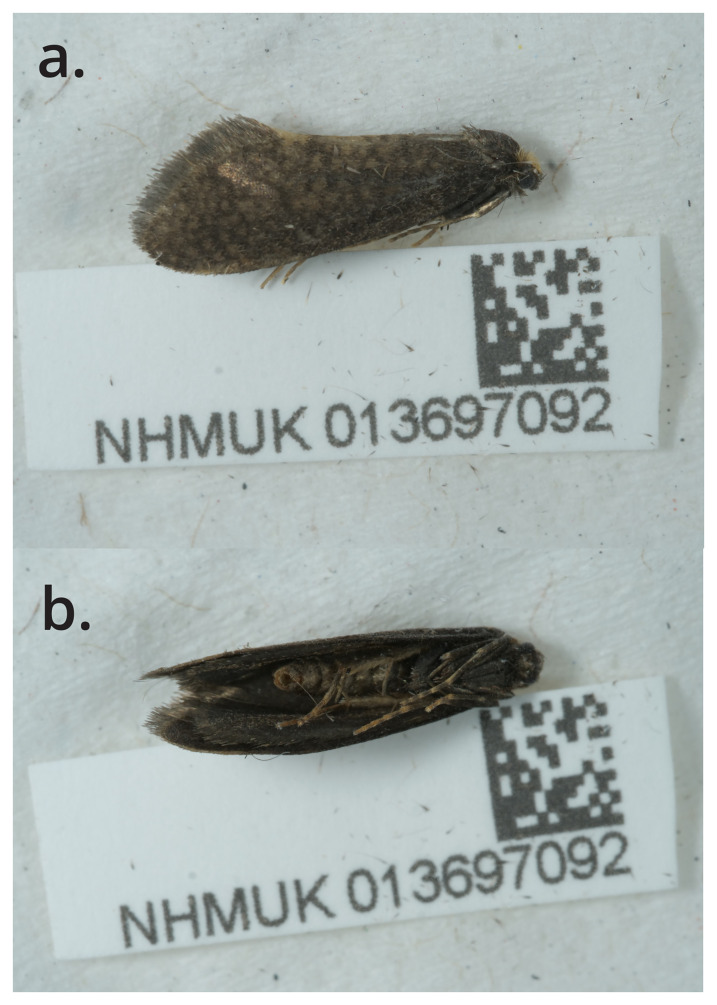
Photographs of the *Taleporia tubulosa* (ilTalTubu1) specimen used for genome sequencing.

The specimen used for genome sequencing was an adult male *Taleporia tubulosa* (specimen ID NHMUK013697092, ToLID ilTalTubu1; Figure 1), collected from Kensington, Wetherby Gardens, England, UK (latitude 51.49, longitude –0.19) on 2022-05-25. The specimen was collected by Maxwell Barclay and identified by David Lees. Details of the sampling and metadata procedures, which followed recommended standards, are described in Lawniczak *et al*. (2022).

The initial identification was verified by an additional DNA barcoding process according to the framework developed by Twyford *et al.* (2024). A small sample was dissected from the specimen and stored in ethanol, while the remaining parts were shipped on dry ice to the Wellcome Sanger Institute (WSI) (see the protocol). The tissue was lysed, the COI marker region was amplified by PCR, and amplicons were sequenced and compared to the BOLD database, confirming the species identification ( Crowley *et al*., 2023). Following whole genome sequence generation, the relevant DNA barcode region was also used alongside the initial barcoding data for sample tracking at the WSI ( Twyford *et al*., 2024). The standard operating procedures for Darwin Tree of Life barcoding are available on protocols.io.

### Nucleic acid extraction

Protocols for high molecular weight (HMW) DNA extraction developed at the Wellcome Sanger Institute (WSI) Tree of Life Core Laboratory are available on protocols.io ( Howard *et al*., 2025). The ilTalTubu1 sample was weighed and triaged to determine the appropriate extraction protocol. Tissue from the whole organism was homogenised by powermashing using a PowerMasher II tissue disruptor.

HMW DNA was extracted in the WSI Scientific Operations core using the Automated MagAttract v2 protocol. DNA was sheared into an average fragment size of 12–20 kb following the Megaruptor®3 for LI PacBio protocol. Sheared DNA was purified by automated SPRI (solid-phase reversible immobilisation). The concentration of the sheared and purified DNA was assessed using a Nanodrop spectrophotometer and Qubit Fluorometer using the Qubit dsDNA High Sensitivity Assay kit. Fragment size distribution was evaluated by running the sample on the FemtoPulse system. For this sample, the final post-shearing DNA had a Qubit concentration of 14.6 ng/μL and a yield of 686.20 ng, with a fragment size of 14.5 kb. The 260/280 spectrophotometric ratio was 1.91, and the 260/230 ratio was 1.22.

### PacBio HiFi library preparation and sequencing

Library preparation and sequencing were performed at the WSI Scientific Operations core. Libraries were prepared using the SMRTbell Prep Kit 3.0 (Pacific Biosciences, California, USA), following the manufacturer’s instructions. The kit includes reagents for end repair/A-tailing, adapter ligation, post-ligation SMRTbell bead clean-up, and nuclease treatment. Size selection and clean-up were performed using diluted AMPure PB beads (Pacific Biosciences). DNA concentration was quantified using a Qubit Fluorometer v4.0 (ThermoFisher Scientific) and the Qubit 1X dsDNA HS assay kit. Final library fragment size was assessed with the Agilent Femto Pulse Automated Pulsed Field CE Instrument (Agilent Technologies) using the gDNA 55 kb BAC analysis kit.

The sample was sequenced on a Revio instrument (Pacific Biosciences). The prepared library was normalised to 2 nM, and 15 μL was used for making complexes. Primers were annealed and polymerases bound to generate circularised complexes, following the manufacturer’s instructions. Complexes were purified using 1.2X SMRTbell beads, then diluted to the Revio loading concentration (200–300 pM) and spiked with a Revio sequencing internal control. The sample was sequenced on a Revio 25M SMRT cell. The SMRT Link software (Pacific Biosciences), a web-based workflow manager, was used to configure and monitor the run and to carry out primary and secondary data analysis.

### Hi-C

***Sample preparation and crosslinking***

The Hi-C sample was prepared from 20–50 mg of frozen tissue from the ilTalTubu1 sample using the Arima-HiC v2 kit (Arima Genomics). Following the manufacturer’s instructions, tissue was fixed and DNA crosslinked using TC buffer to a final formaldehyde concentration of 2%. The tissue was homogenised using the Diagnocine Power Masher-II. Crosslinked DNA was digested with a restriction enzyme master mix, biotinylated, and ligated. Clean-up was performed with SPRISelect beads before library preparation. DNA concentration was measured with the Qubit Fluorometer (Thermo Fisher Scientific) and Qubit HS Assay Kit. The biotinylation percentage was estimated using the Arima-HiC v2 QC beads.

***Hi-C library preparation and sequencing***

Biotinylated DNA constructs were fragmented using a Covaris E220 sonicator and size selected to 400–600 bp using SPRISelect beads. DNA was enriched with Arima-HiC v2 kit Enrichment beads. End repair, A-tailing, and adapter ligation were carried out with the NEBNext Ultra II DNA Library Prep Kit (New England Biolabs), following a modified protocol where library preparation occurs while DNA remains bound to the Enrichment beads. Library amplification was performed using KAPA HiFi HotStart mix and a custom Unique Dual Index (UDI) barcode set (Integrated DNA Technologies). Depending on sample concentration and biotinylation percentage determined at the crosslinking stage, libraries were amplified with 10–16 PCR cycles. Post-PCR clean-up was performed with SPRISelect beads. Libraries were quantified using the AccuClear Ultra High Sensitivity dsDNA Standards Assay Kit (Biotium) and a FLUOstar Omega plate reader (BMG Labtech).

Prior to sequencing, libraries were normalised to 10 ng/μL. Normalised libraries were quantified again and equimolar and/or weighted 2.8 nM pools. Pool concentrations were checked using the Agilent 4200 TapeStation (Agilent) with High Sensitivity D500 reagents before sequencing. Sequencing was performed using paired-end 150 bp reads on the Illumina NovaSeq X.

### Genome assembly

Prior to assembly of the PacBio HiFi reads, a database of *k*-mer counts ( *k* = 31) was generated from the filtered reads using FastK. GenomeScope2 ( Ranallo-Benavidez *et al*., 2020) was used to analyse the *k*-mer frequency distributions, providing estimates of genome size, heterozygosity, and repeat content.

The HiFi reads were assembled using Hifiasm in Hi-C phasing mode ( Cheng *et al*., 2021; Cheng *et al*., 2022), producing two haplotypes. Hi-C reads ( Rao *et al*., 2014) were mapped to the primary contigs using bwa-mem2 ( Vasimuddin *et al*., 2019). Contigs were further scaffolded with Hi-C data in YaHS ( Zhou *et al*., 2023), using the --break option for handling potential misassemblies. The scaffolded assemblies were evaluated using Gfastats ( Formenti *et al*., 2022), BUSCO ( Manni *et al*., 2021) and MERQURY.FK ( Rhie *et al*., 2020).

The mitochondrial genome was assembled using MitoHiFi ( Uliano-Silva *et al*., 2023), which runs MitoFinder ( Allio *et al*., 2020) and uses these annotations to select the final mitochondrial contig and to ensure the general quality of the sequence.

### Assembly curation

The assembly was decontaminated using the Assembly Screen for Cobionts and Contaminants ( ASCC) pipeline. TreeVal was used to generate the flat files and maps for use in curation. Manual curation was conducted primarily in PretextView and HiGlass ( Kerpedjiev *et al*., 2018). Scaffolds were visually inspected and corrected as described by Howe *et al*. (2021). Manual corrections included 77 breaks and 153 joins. The curation process is documented at https://gitlab.com/wtsi-grit/rapid-curation. PretextSnapshot was used to generate a Hi-C contact map of the final assembly.

### Assembly quality assessment

The Merqury.FK tool ( Rhie *et al*., 2020) was run in a Singularity container ( Kurtzer *et al*., 2017) to evaluate *k*-mer completeness and assembly quality for both haplotypes using the *k*-mer databases ( *k* = 31) computed prior to genome assembly. The analysis outputs included assembly QV scores and completeness statistics.

The genome was analysed using the BlobToolKit pipeline, a Nextflow implementation of the earlier Snakemake version ( Challis *et al*., 2020). The pipeline aligns PacBio reads using minimap2 ( Li, 2018) and SAMtools ( Danecek *et al*., 2021) to generate coverage tracks. It runs BUSCO ( Manni *et al*., 2021) using lineages identified from the NCBI Taxonomy ( Schoch *et al*., 2020). For the three domain-level lineages, BUSCO genes are aligned to the UniProt Reference Proteomes database ( Bateman *et al*., 2023) using DIAMOND blastp ( Buchfink *et al*., 2021). The genome is divided into chunks based on the density of BUSCO genes from the closest taxonomic lineage, and each chunk is aligned to the UniProt Reference Proteomes database with DIAMOND blastx. Sequences without hits are chunked using seqtk and aligned to the NT database with blastn ( Altschul *et al*., 1990). The BlobToolKit suite consolidates all outputs into a blobdir for visualisation. The BlobToolKit pipeline was developed using nf-core tooling ( Ewels *et al*., 2020) and MultiQC ( Ewels *et al*., 2016), with containerisation through Docker ( Merkel, 2014) and Singularity ( Kurtzer *et al*., 2017).

## Genome sequence report

### Sequence data

**Figure 2.**
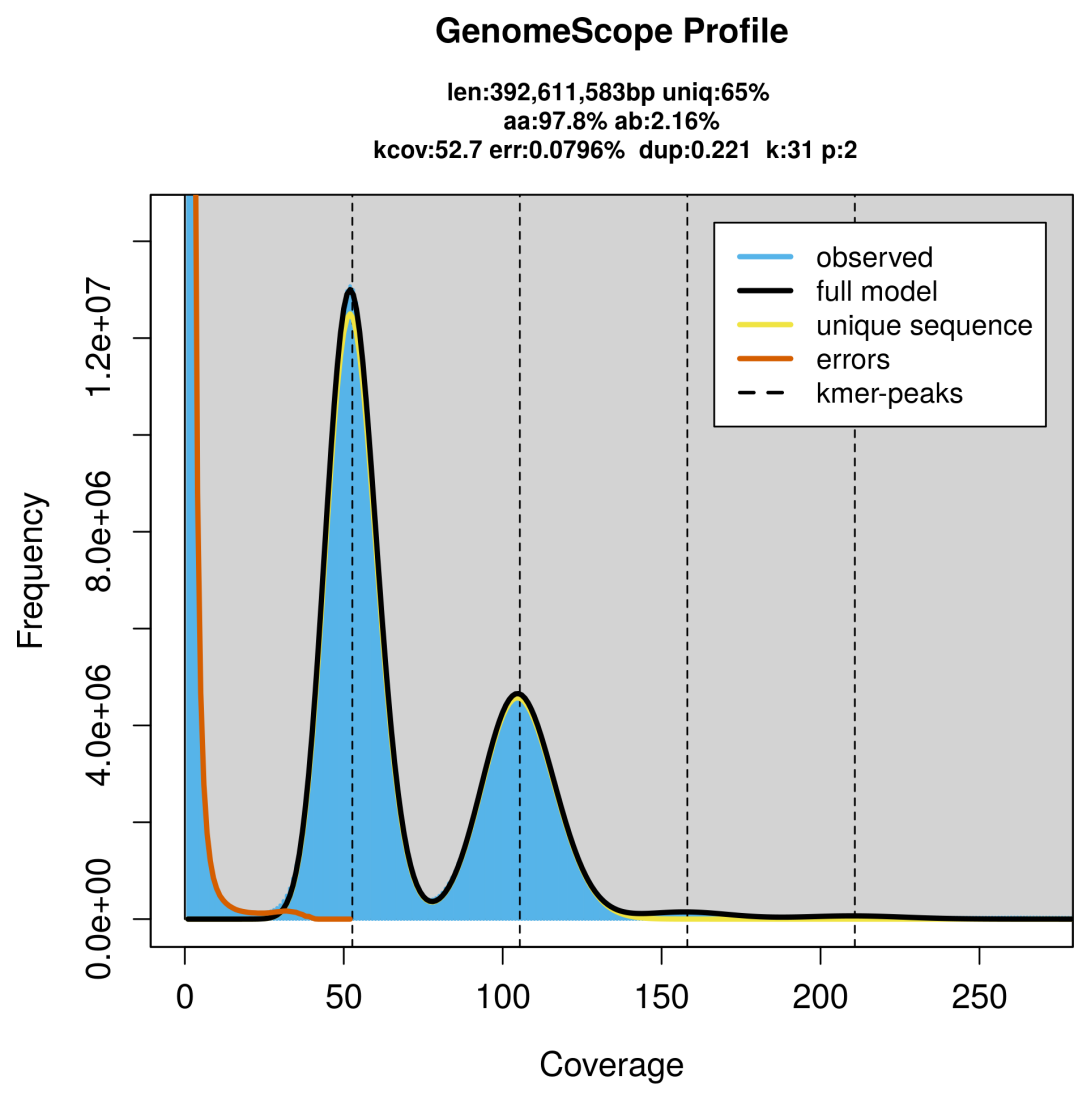
Frequency distribution of *k*-mers generated using GenomeScope2. The plot shows observed and modelled *k*-mer spectra, providing estimates of genome size, heterozygosity, and repeat content based on unassembled sequencing reads.

PacBio sequencing of the *Taleporia tubulosa* specimen generated 42.56 Gb (gigabases) from 4.77 million reads, which were used to assemble the genome. GenomeScope2.0 analysis estimated the haploid genome size at 392.61 Mb, with a heterozygosity of 2.16% and repeat content of 35.00% ( Figure 2). These estimates guided expectations for the assembly. Based on the estimated genome size, the sequencing data provided approximately 105× coverage. Hi-C sequencing produced 93.14 Gb from 616.84 million reads, which were used to scaffold the assembly. Table 1 summarises the specimen and sequencing details.

**Table 1.** Specimen and sequencing data for BioProject PRJEB81656.

Platform	PacBio HiFi	Hi-C
**ToLID**	ilTalTubu1	ilTalTubu1
**Specimen ID**	NHMUK013697092	NHMUK013697092
**BioSample (source individual)**	SAMEA115574765	SAMEA115574765
**BioSample (tissue)**	SAMEA115599864	SAMEA115599864
**Tissue**	whole organism	whole organism
**Instrument**	Revio	Illumina NovaSeq X
**Run accessions**	ERR13900477	ERR13907251
**Read count total**	4.77 million	616.84 million
**Base count total**	42.56 Gb	93.14 Gb

### Assembly statistics

The genome was assembled into two haplotypes using Hi-C phasing. Haplotype 1 was curated to chromosome level, while haplotype 2 was assembled to scaffold level. The final assembly has a total length of 394.58 Mb in 61 scaffolds, with 117 gaps, and a scaffold N50 of 14.43 Mb ( Table 2).

**Table 2.** Genome assembly statistics.

**Assembly name**	ilTalTubu1.hap1.1	ilTalTubu1.hap2.1
**Assembly accession**	GCA_964330275.1	GCA_964330315.1
**Assembly level**	chromosome	scaffold
**Span (Mb)**	394.58	397.58
**Number of chromosomes**	30	Scaffold-level
**Number of contigs**	178	225
**Contig N50**	6.98 Mb	6.36 Mb
**Number of scaffolds**	61	90
**Scaffold N50**	14.43 Mb	14.63 Mb
**Longest scaffold length (Mb)**	23.41	-
**Sex chromosomes**	Z	-
**Organelles**	Mitochondrion: 17.11 kb	-

**Figure 3.**
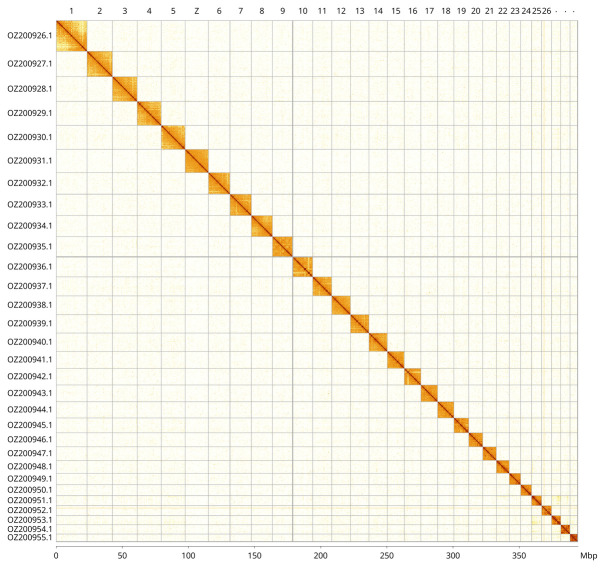
Hi-C contact map of the *Taleporia tubulosa* genome assembly. Assembled chromosomes are shown in order of size and labelled along the axes, with a megabase scale shown below. The plot was generated using PretextSnapshot.

Most of the assembly sequence (99.89%) was assigned to 30 chromosomal-level scaffolds, representing 29 autosomes and the Z sex chromosome. These chromosome-level scaffolds, confirmed by Hi-C data, are named according to size ( Figure 3; Table 3). Chromosome Z was identified through the detection of ancestral BUSCO genes ( Wright *et al*., 2024).

**Table 3.** Chromosomal pseudomolecules in the haplotype 1 genome assembly of *Taleporia tubulosa* ilTalTubu1.

INSDC accession	Molecule	Length (Mb)	GC%
OZ200926.1	1	23.41	38
OZ200927.1	2	19.23	38
OZ200928.1	3	18.64	37.50
OZ200929.1	4	18.25	38
OZ200930.1	5	18.08	38
OZ200932.1	6	16.40	37.50
OZ200933.1	7	16.08	37.50
OZ200934.1	8	15.95	37.50
OZ200935.1	9	15.50	37.50
OZ200936.1	10	14.90	37.50
OZ200937.1	11	14.43	37.50
OZ200938.1	12	14.17	38
OZ200939.1	13	13.93	37.50
OZ200940.1	14	13.91	38
OZ200941.1	15	12.80	37.50
OZ200942.1	16	12.63	38
OZ200943.1	17	12.62	37.50
OZ200944.1	18	12.29	37.50
OZ200945.1	19	11.22	38
OZ200946.1	20	10.55	37.50
OZ200947.1	21	10.33	37.50
OZ200948.1	22	9.70	38
OZ200949.1	23	8.67	37.50
OZ200950.1	24	8.25	37.50
OZ200951.1	25	7.61	39.50
OZ200952.1	26	7.44	38
OZ200953.1	27	7.06	38.50
OZ200954.1	28	6.90	38.50
OZ200955.1	29	5.69	38.50
OZ200931.1	Z	17.50	37.50

The mitochondrial genome was also assembled (length 17.11 kb, OZ200956.1). This sequence is included as a contig in the multifasta file of the genome submission and as a standalone record.

**Figure 4.**
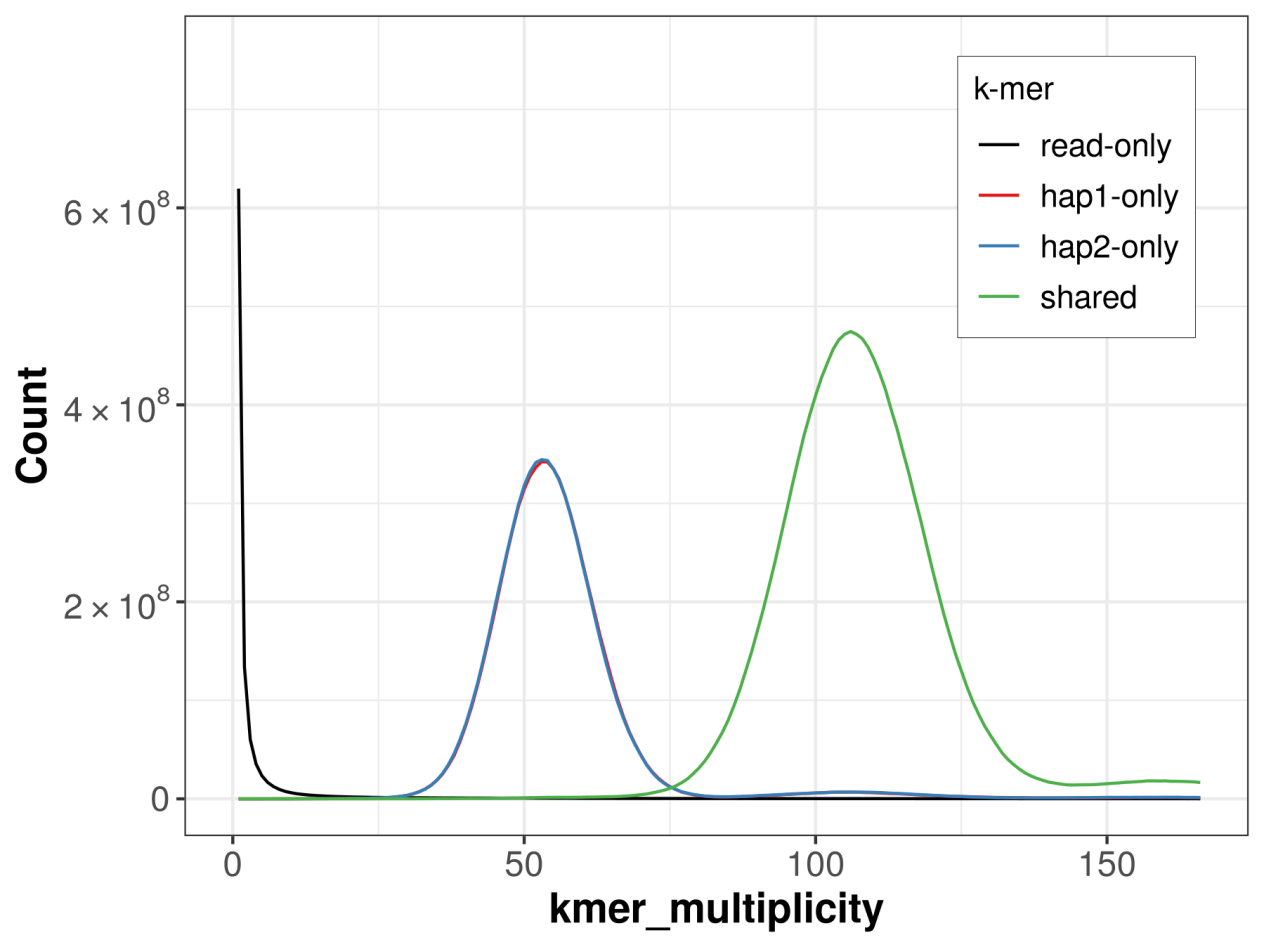
Evaluation of *k*-mer completeness using MerquryFK. This plot illustrates the recovery of *k*‐mers from the original read data in the final assemblies. The horizontal axis represents *k*‐mer multiplicity, and the vertical axis shows the number of *k*‐mers. The black curve represents *k*‐mers that appear in the reads but are not assembled. The green curve corresponds to *k*‐mers shared by both haplotypes, and the red and blue curves show *k*‐mers found only in one of the haplotypes.

For haplotype 1, the estimated QV is 64.1, and for haplotype 2, 64.0. When the two haplotypes are combined, the assembly achieves an estimated QV of 64.1. The *k*-mer completeness is 67.45% for haplotype 1, 67.61% for haplotype 2, and 99.69% for the combined haplotypes ( Figure 4).

**Figure 5.**
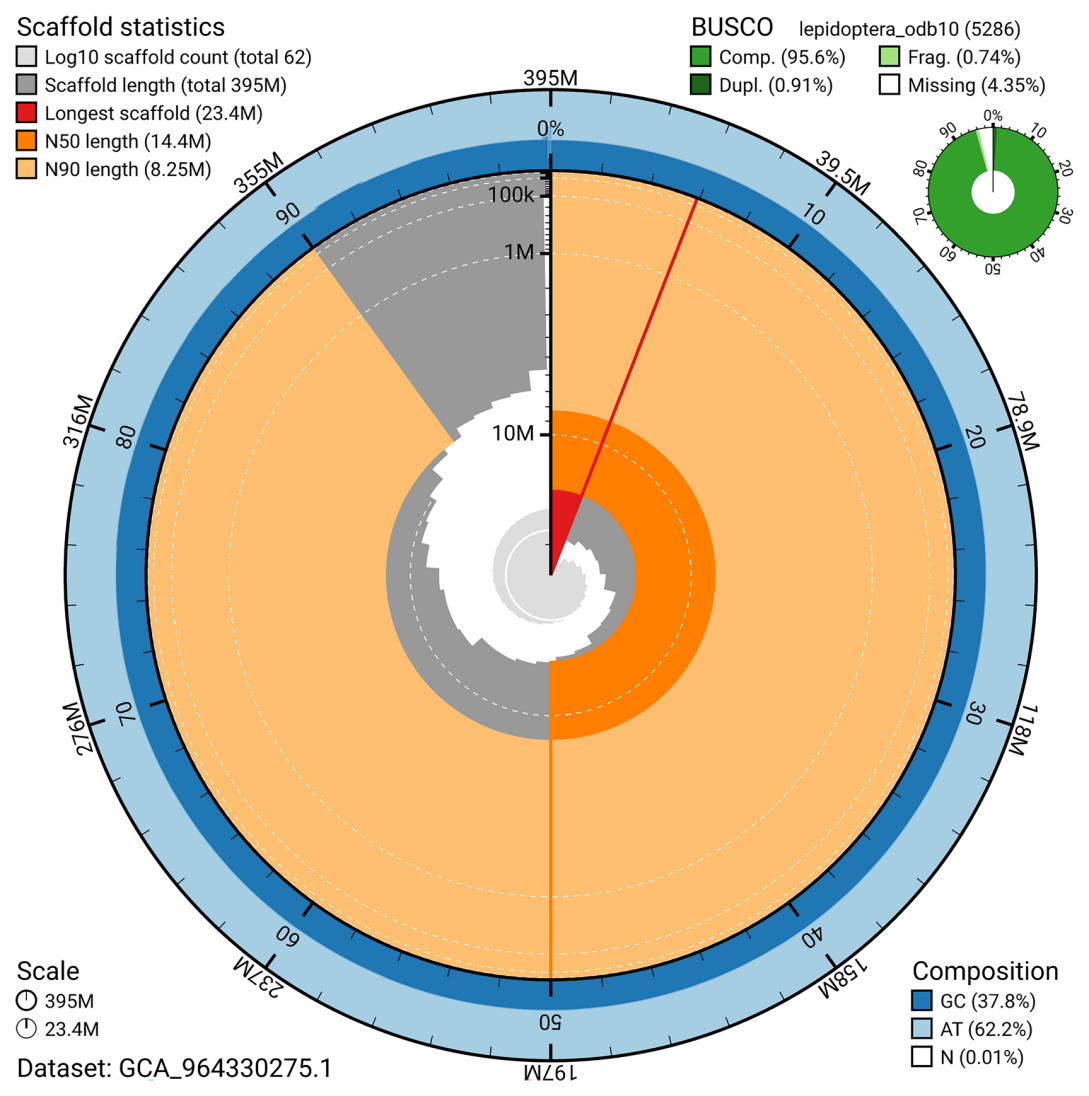
Assembly metrics for ilTalTubu1.hap1.1. The BlobToolKit snail plot provides an overview of assembly metrics and BUSCO gene completeness. The circumference represents the length of the whole genome sequence, and the main plot is divided into 1 000 bins around the circumference. The outermost blue tracks display the distribution of GC, AT, and N percentages across the bins. Scaffolds are arranged clockwise from longest to shortest and are depicted in dark grey. The longest scaffold is indicated by the red arc, and the deeper orange and pale orange arcs represent the N50 and N90 lengths. A light grey spiral at the centre shows the cumulative scaffold count on a logarithmic scale. A summary of complete, fragmented, duplicated, and missing BUSCO genes in the set is presented at the top right. An interactive version of this figure can be accessed on the BlobToolKit viewer.

**Figure 6.**
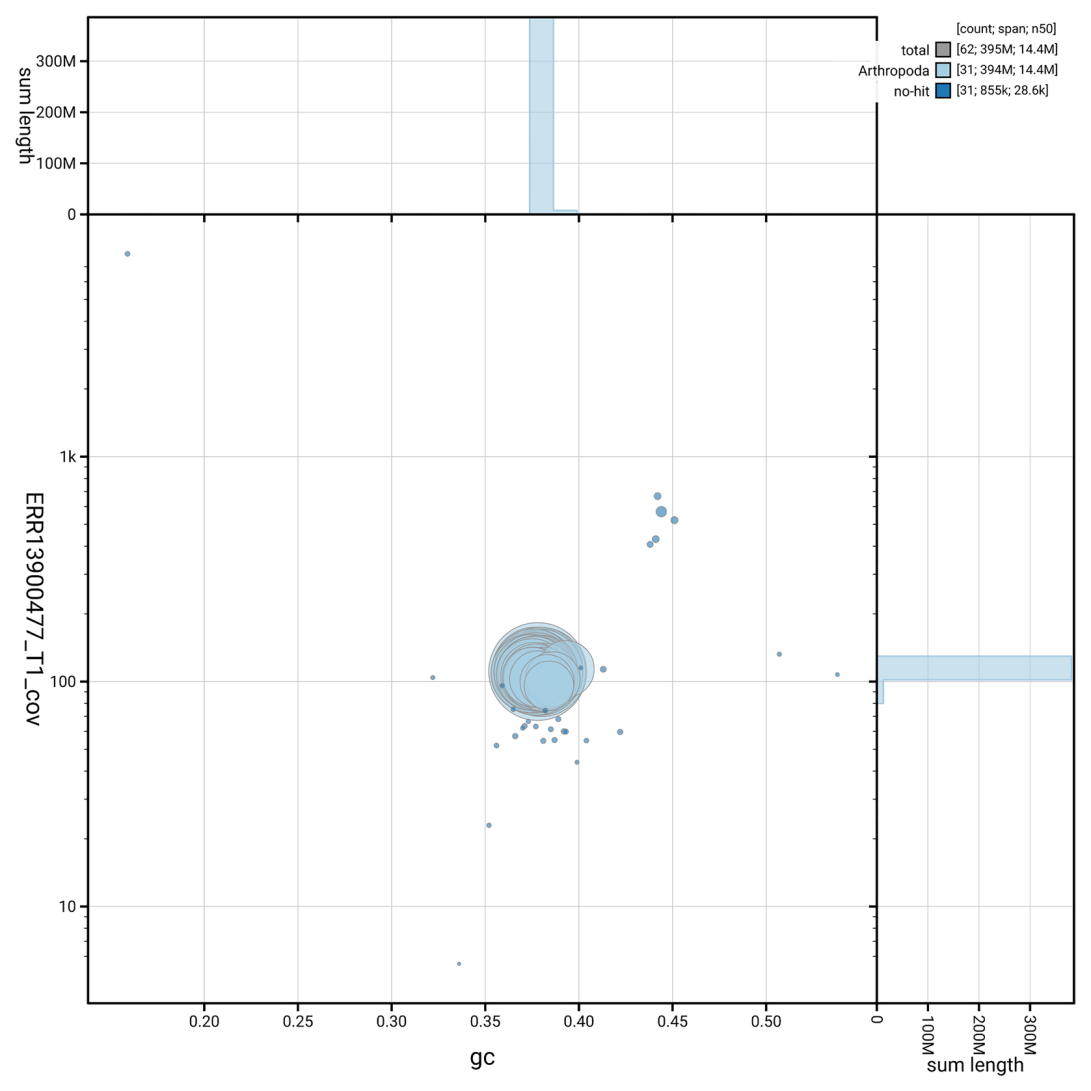
BlobToolKit GC-coverage plot for ilTalTubu1.hap1.1. Blob plot showing sequence coverage (vertical axis) and GC content (horizontal axis). The circles represent scaffolds, with the size proportional to scaffold length and the colour representing phylum membership. The histograms along the axes display the total length of sequences distributed across different levels of coverage and GC content. An interactive version of this figure is available on the BlobToolKit viewer.

BUSCO analysis using the lepidoptera_odb10 reference set ( *n* = 5 286) identified 95.6% of the expected gene set (single = 94.7%, duplicated = 0.9%) for haplotype 1. The snail plot in Figure 5 summarises the scaffold length distribution and other assembly statistics for haplotype 1. The blob plot in Figure 6 shows the distribution of scaffolds by GC proportion and coverage for haplotype 1.

Table 4 lists the assembly metric benchmarks adapted from Rhie *et al.* (2021) and the Earth BioGenome Project Report on Assembly Standards September 2024. The EBP metric, calculated for the haplotype 1, is **6.C.Q64**, meeting the recommended reference standard.

**Table 4.** Earth Biogenome Project summary metrics for the *Taleporia tubulosa* assembly.

Measure	Value	Benchmark
EBP summary (haplotype 1)	6.C.Q64	6.C.Q40
Contig N50 length	6.98 Mb	≥ 1 Mb
Scaffold N50 length	14.43 Mb	= chromosome N50
Consensus quality (QV)	Haplotype 1: 64.1; haplotype 2: 64.0; combined: 64.1	≥ 40
*k*-mer completeness	Haplotype 1: 67.45%; Haplotype 2: 67.61%; combined: 99.69%	≥ 95%
BUSCO	C:95.6% [S:94.7%; D:0.9%]; F:0.7%; M:3.6%; n:5 286	S > 90%; D < 5%
Percentage of assembly assigned to chromosomes	99.89%	≥ 90%

### Wellcome Sanger Institute – Legal and Governance

The materials that have contributed to this genome note have been supplied by a Darwin Tree of Life Partner. The submission of materials by a Darwin Tree of Life Partner is subject to the **‘Darwin Tree of Life Project Sampling Code of Practice’**, which can be found in full on the Darwin Tree of Life website. By agreeing with and signing up to the Sampling Code of Practice, the Darwin Tree of Life Partner agrees they will meet the legal and ethical requirements and standards set out within this document in respect of all samples acquired for, and supplied to, the Darwin Tree of Life Project. Further, the Wellcome Sanger Institute employs a process whereby due diligence is carried out proportionate to the nature of the materials themselves, and the circumstances under which they have been/are to be collected and provided for use. The purpose of this is to address and mitigate any potential legal and/or ethical implications of receipt and use of the materials as part of the research project, and to ensure that in doing so we align with best practice wherever possible. The overarching areas of consideration are:

- Ethical review of provenance and sourcing of the material
- Legality of collection, transfer and use (national and international)

Each transfer of samples is further undertaken according to a Research Collaboration Agreement or Material Transfer Agreement entered into by the Darwin Tree of Life Partner, Genome Research Limited (operating as the Wellcome Sanger Institute), and in some circumstances, other Darwin Tree of Life collaborators.

## Data Availability

European Nucleotide Archive: Taleporia tubulosa. Accession number PRJEB81656. The genome sequence is released openly for reuse. The *Taleporia tubulosa* genome sequencing initiative is part of the Darwin Tree of Life Project (PRJEB40665), the Sanger Institute Tree of Life Programme (PRJEB43745) and Project Psyche (PRJEB71705). All raw sequence data and the assembly have been deposited in INSDC databases. The genome will be annotated using available RNA-Seq data and presented through the Ensembl pipeline at the European Bioinformatics Institute. Raw data and assembly accession identifiers are reported in Table 1 and Table 2. Production code used in genome assembly at the WSI Tree of Life is available at https://github.com/sanger-tol. Table 5 lists software versions used in this study.

## Author information

Contributors are listed at the following links:

Members of the Natural History Museum Genome Acquisition Lab

Members of the Darwin Tree of Life Barcoding collective

Members of the Wellcome Sanger Institute Tree of Life Management, Samples and Laboratory team

Members of Wellcome Sanger Institute Scientific Operations – Sequencing Operations

Members of the Wellcome Sanger Institute Tree of Life Core Informatics team

Members of the Tree of Life Core Informatics collective

Members of the Darwin Tree of Life Consortium
