# Supplementary material for: The genome sequence of the Brown Bagworm, Taleporia tubulosa (Retzius, 1783) (Lepidoptera: Psychidae)
Source: Wellcome Open Res. 2025 Oct 21;10:589. [Version 1] doi: 10.12688/wellcomeopenres.25052.1 (PMC12743216; doi:10.12688/wellcomeopenres.25052.1)
Supplement: Peer review history

# Reviewer report: Reviewer response for version 1

**DOI:** 10.21956/wellcomeopenres.27618.r141729

**Published:** 2026-01-22

**Authors:** Marta Vila

**Affiliations:** 1 Universidade da Coruna, Galicia, Spain

This is a fine genome note produced by members of the Darwin Tree of Life Consortium. 

 My only suggestion is to revise the writing of the second to last paragraph of the Background section.

 (1) Readers without a deep knowledge of the Lepidopteran phylogeny might get confused when reading the first sentence.

 (2) In my opinion, the sentence" "Females lack sex chromatin, have a lower chromosome number and smaller genome size than males" can be deleted.

 (3) I find the sentence "Instead, Psychidae show a ZO/ZZ sex chromosome system" somehow disconnected from the previous info. 

 I hope this helps. Congratulations for this work.

Are sufficient details of methods and materials provided to allow replication by others?

Yes

Is the rationale for creating the dataset(s) clearly described?

Yes

Are the datasets clearly presented in a useable and accessible format?

Yes

Are the protocols appropriate and is the work technically sound?

Yes

Reviewer Expertise:

Lepidoptera, Phylogeography, Molecular Ecology, Genomics

I confirm that I have read this submission and believe that I have an appropriate level of expertise to confirm that it is of an acceptable scientific standard.

# Reviewer report: Reviewer response for version 1

**DOI:** 10.21956/wellcomeopenres.27618.r141724

**Published:** 2025-12-27

**Authors:** Jason Charamis

**Affiliations:** 1 Foundation for Research and Technology - Hellas, Irákleion, Greece

This Data Note successfully presents a high-quality, chromosome-level reference genome for the Brown Bagworm, *Taleporia tubulosa*, produced through the Darwin Tree of Life project using PacBio HiFi and Hi-C sequencing. The assembly exceeds Earth BioGenome Project standards, scaffolding 99.89% of the sequence into 30 pseudomolecules, including the Z chromosome, with a high consensus quality. By documenting the assembly of two haplotypes and the mitochondrial genome, the manuscript effectively contextualizes this resource to support future evolutionary studies on winglessness and sex chromosomes. The work constitutes a technically sound and valuable contribution to biodiversity genomics.

Are sufficient details of methods and materials provided to allow replication by others?

Yes

Is the rationale for creating the dataset(s) clearly described?

Yes

Are the datasets clearly presented in a useable and accessible format?

Yes

Are the protocols appropriate and is the work technically sound?

Yes

Reviewer Expertise:

Arthropod Genomics

I confirm that I have read this submission and believe that I have an appropriate level of expertise to confirm that it is of an acceptable scientific standard.

# Reviewer report: Reviewer response for version 1

**DOI:** 10.21956/wellcomeopenres.27618.r136940

**Published:** 2025-11-14

**Authors:** Shiqi Luo

**Affiliations:** 1 South China Agricultural University, Guangzhou, Guangdong, China

This article sequenced a male individual of the Brown Bagworm *Taleporia tubulosa* and assembled a chromosome-level genome, employing various sequencing technologies such as Pac-Bio long-read sequencing and Hi-C. The overall assembly quality is high. The study utilized standardized experimental and analytical methods, ensuring high data credibility and reproducibility. The data storage information is clearly described, facilitating access and use by other interested researchers.

 A few suggestions:

1. In the introduction, the authors said: "The genome will be useful for further studies in lepidopteran chromosome evolution, as well as studies of winglessness (e.g., Niitsu et al., 2017). This detailed phylogenetic study based on 28S rRNA and reconstructing patterns of wing degeneration in the family (Niitsu et al., 2017) recovered *Taleporia* as sister to the genus *Kozhantshikovia* Saigusa, 1961 and the subfamily Taleporiinae as sister to Narycinae." These two sentences could be revised for greater clarity.
2. When using relevant software, if parameters other than the default values were used, it is recommended to provide more specific parameter information.
3. The chromosome numbers on the x-axis of Figure 3 should be fully displayed.

Are sufficient details of methods and materials provided to allow replication by others?

Partly

Is the rationale for creating the dataset(s) clearly described?

Yes

Are the datasets clearly presented in a useable and accessible format?

Yes

Are the protocols appropriate and is the work technically sound?

Yes

Reviewer Expertise:

functional and evolutionary genomics, honey bee

I confirm that I have read this submission and believe that I have an appropriate level of expertise to confirm that it is of an acceptable scientific standard.
